# Characterizing tobacco control mass media campaigns in England

**DOI:** 10.1111/add.12293

**Published:** 2013-08-14

**Authors:** Tessa Langley, Sarah Lewis, Ann McNeill, Anna Gilmore, Lisa Szatkowski, Robert West, Michelle Sims

**Affiliations:** 1 UK Centre for Tobacco Control Studies, Division of Epidemiology and Public Health, University of NottinghamNottingham, UK; 2 UK Centre for Tobacco Control Studies, National Addiction Centre, Institute of Psychiatry, King's College LondonLondon, UK; 3 UK Centre for Tobacco Control Studies, Department for Health, University of BathBath, UK; 4 UK Centre for Tobacco Control Studies, Health Behaviour Research Centre, University College LondonLondon, UK

**Keywords:** Mass media campaigns, public spending, tobacco control

## Abstract

**Aims** To characterize publically funded tobacco control campaigns in England between 2004 and 2010 and to explore if they were in line with recommendations from the literature in terms of their content and intensity. International evidence suggests that campaigns which warn of the negative consequences of smoking and feature testimonials from real-life smokers are most effective, and that four exposures per head per month are required to reduce smoking prevalence. **Design** Characterization of tobacco control advertisements using a theoretically based framework designed to describe advertisement themes, informational and emotional content and style. Study of the intensity of advertising and exposure to different types of advertisement using data on population-level exposure to advertisements shown during the study period. **Setting** England. **Measurements** Television Ratings (TVRs), a standard measure of advertising exposure, were used to calculate exposure to each different campaign type. **Findings** A total of 89% of advertising was for smoking cessation; half of this advertising warned of the negative consequences of smoking, while half contained how-to-quit messages. Acted scenes featured in 72% of advertising, while only 17% featured real-life testimonials. Only 39% of months had at least four exposures to tobacco control campaigns per head. **Conclusions** A theory-driven approach enabled a systematic characterization of tobacco control advertisements in England. Between 2004 and 2010 only a small proportion of tobacco control advertisements utilized the most effective strategies—negative health effects messages and testimonials from real-life smokers. The intensity of campaigns was lower than international recommendations.

## Introduction

International evidence suggests that tobacco control mass media campaigns (MMCs) can promote adult quitting and reduce smoking prevalence, although their effectiveness depends on the type and intensity of campaign 1.

Campaigns differ in theme (e.g. smoking cessation, passive smoking), purpose (e.g. informing people about methods of quitting, or the negative health effects of smoking), emotional tone and style (e.g. acted scenes, testimonials). A recent review concluded that negative health effects messages which emphasize the serious health effects of smoking for individual and/or family or friends are generally more effective than how-to-quit messages providing information about effective methods of smoking cessation or anti-industry advertisements 1. It also found that advertisements with high emotional content and testimonial advertisements (both of which tend to contain negative health effects messages) are most effective at increasing quit rates. The review identified a key challenge associated with untangling the effective elements of negative health effects messages, as particular elements (negative health effects information, graphic/testimonial formats and high levels of negative emotions) often co-occur. It is not clear whether how-to-quit and anti-industry messages which feature high levels of emotion and testimonials could be similarly effective as the most effective negative health effects messages.

Campaign intensity is measured in Television Ratings (TVRs, also known as Gross Rating Points, or GRPs), a standard industry measure of campaign reach multiplied by frequency. For example, 500 TVRs equates to, on average, 100% of people within a region being exposed an advertisement five times, or 50% of people being exposed to the advertisement 10 times. Wakefield *et al*. conducted a time–series analysis of mass media campaign exposure data which suggested that 400 TVRs per month are needed to reduce smoking prevalence 2. The US Centers for Disease Control and Prevention also recommends 1200 TVRs per quarter in its Best Practice guidelines 3. In addition, the Wakefield study highlighted that sustained behaviour change requires sustained campaign exposure due to the short-lived effects of campaigns: the impact of mass media campaigns on smoking prevalence was estimated to last just 2 months 2. A further study, which investigated the impact of tobacco control campaigns in a cohort of smokers, found that their impact on quit attempts lasts 3 months 4.

Although MMCs are a costly element of the highly comprehensive framework of tobacco control interventions in the United Kingdom, there is very little evidence relating to their effectiveness in the United Kingdom and none examining the characteristics (i.e. typology and intensity) of campaigns that determine their effectiveness. Such knowledge is important both to ensure maximal cost-effectiveness of such campaigns, but also because it will be important in interpreting evaluations of the effectiveness of MMCs in the United Kingdom. To our knowledge, there has only been one peer-reviewed study of the effectiveness of campaigns in the United Kingdom in the past decade 5. This study found that, in England, a 1% increase in TVRs increased calls to the national quitline by 0.085%.

This study therefore aims to characterize campaigns funded and run by the Department of Health in England between 2004 and 2010 in terms of their themes, informational content, emotional content, style and intensity, and to explore whether or not they were in line with recommendations from the literature in terms of their content and intensity. In particular, the study focuses on whether any important elements were absent or underused and thus whether MMCs in the United Kingdom are likely to have been maximally effective.

## Methods

### Tobacco control advertisement coding framework

We used a theory-driven approach to develop a framework to characterize tobacco control advertisements on television. The broad theoretical model of behaviour we have utilized is the COM-B, which provides a way of determining the key constructs required to change behaviour: capability, opportunity, and/or motivation 6. The key construct that advertisements should be addressing is motivation, and the model of motivation we used was the PRIME theory (plans, responses, impulses/inhibitory forces, motives and evaluations) 7. This theory recognizes that ‘at every moment we act in pursuit of what we most want or need at that moment’. ‘Want’ arises from anticipated pleasure or satisfaction and ‘need’ arises from anticipated relief from mental or physical discomfort. The focus on wants and needs is important, because it recognizes that emotion and drive states are the fundamental driver of behaviour change. In addition, in order for the emotion to generate a want or need to stop smoking, the smoker must have a clear action plan and realistic hope that the action will be successful in order to generate the anticipated pleasure, satisfaction or relief. After a quit attempt has started, for it to be sustained the want or need to refrain from smoking must be greater than the want or need to smoke at all times when smoking is a possibility. Identity (our mental representations of ourselves and feelings attached to these) is recognized to be an important source of wants and needs 7. For example, ‘liking’ being a smoker has been found to be an important barrier to quit attempts and developing an identity as someone who is trying to stop or an ex-smoker is important in promoting cessation activity 8. The focus on the moment is also important because it recognizes that decisions to take action in the future can only influence that action if they are remembered at the time, the individual is still committed to them, and the want or need that they generate is sufficiently strong to overcome wants or needs present at the time. Prompting immediate action may therefore be important.

The advertisement coding framework was developed based on the PRIME theory tenets outlined above, and was refined by a group of five researchers, who coded 12 advertisements independently to establish whether the framework was clear, included all the key elements of the advertisements, and whether or not there was broad concordance in coding. The framework is shown in Table 1.

**Table 1:** Framework for categorization of mass media campaigns

Key theme Smoking cessation (in adults) Smoking cessation (in adolescents) Preventing uptake (in adults) Preventing uptake (in adolescents) Smoking in pregnancy (anyone smoking around unborn) Exposure to and effects of passive smoking Smoke-free legislation (smoking in public places) Anti-industry Informational content (implicit/explicit) Provide information on the negative consequences of smoking (e.g. negative health effects messages about damage to own health and wellbeing and damage to others' health and wellbeing from passive smoking, immoral behaviour of tobacco companies, stigma of smoking) Provide information on the positive consequences of smoking cessation/other smoking behaviour change (e.g. benefits to own health and wellbeing, financial benefit of stopping, improved social standing) Provide information on how to quit (e.g. messages about medication, quitline, local stop smoking services, website, health professional, social support) Emotional content Use imagery to evoke negative feelings about smoking Use positive imagery to evoke positive feelings about quitting Style of delivery Acted scenes Testimonials Graphical aids (including visual depictions of evidence such as graphs, or images of diseased body parts) Cartoons Music Child featured in advertisement—in person or picture

### Coding of advertisement creatives

We obtained advertisement creatives from the Central Office of Information (COI, the UK government's marketing and communications agency, which closed in March 2012) and the Department of Health Tobacco Marketing Team and categorized all government-run tobacco control advertisements televised between 2004 and April 2010, after which campaigns were suspended for 18 months. These campaigns were funded by the Department of Health and were therefore run primarily in England, but were also shown in parts of Wales due to overlapping TV regions. Two researchers categorized the advertisements independently. There was complete concordance on theme, emotional content and style. A third researcher helped to resolve disagreement about the informational content of one advertisement; there was concordance on all others. Ninety-four advertisements were coded. Table 2 provides examples of how the advertisements were coded. Multiple answers could be given for informational content, emotional content and style of delivery.

**Table 2:** Examples of how advertisements were categorized

Campaign	Coding	Advertisement description
Emotional consequences (Anthony Hicks) 14	Theme: smoking cessation	Featured Anthony Hicks, a 58-year-old smoker with throat and lung cancer, lying in a hospital bed struggling to breathe. Mr Hicks talks about his illness and how his daughter is due to visit him. The following image says he died 10 days after filming, and never got to see his daughter again
	Informational content: negative health and emotional consequences of smoking	
	Emotional content: negative	
	Style: testimonial	
Reasons 15	Theme: smoking cessation	Featured a number of adults talking about their reasons for giving up smoking. Advertisement showed parents talking about all the things they're looking forward to doing with their children. These included weddings; teaching their kids to drive; special holidays; and holding their grandchildren
	Informational content: positive consequences of quitting, how to quit	
	Emotional content: positive	
	Style: acted scenes	

We used a focus group of smokers to validate both the framework and our coding, to explore any differences between their coding of the advertisements and our own. The members of the focus group were a subset of eight members of the UK Centre for Tobacco Control Studies' Smokers Panel. This panel is run at the University of Bath, which meets twice a year to discuss issues surrounding smoking, including smoking cessation and reducing uptake. We showed them 12 advertisements, which varied in terms of theme, content and style. They coded the advertisements using the framework and discussed their responses to them. The focus group suggested that the framework contains all the key elements related to theme, content and style. There were no major discrepancies between participants' coding and our own.

We also used the focus group to explore whether the advertisements could also be coded for specific emotional response (e.g. anger, fear, disgust, happiness, satisfaction). While all responses were consistent in terms of whether advertisements evoked positive or negative emotional responses, there was substantial variation in the specific emotion evoked (for example, fear, sadness or guilt, happiness or satisfaction). In our characterization, therefore, emotional content is categorized only as positive or negative.

### Measures of exposure to campaign types

We obtained TVR data summarized by creative and by month for England for each government-run advertisement between 2004 and 2010 from COI. To ensure the validity of these data, we compared these data with other documents containing TVR data, including annual marketing plans from COI. We used the TVR data to calculate monthly intensity of exposure to MMCs overall, calculating the proportion of months where TVRs reached more than 400, the estimated number of TVRs required per month to reduce smoking prevalence 1. We then examined the number of TVRs for each different MMC type (categorized by theme, content and style of campaign) during the whole study period.

In addition, due to survey data cited in the recent Department of Health Smokefree Marketing Campaign Strategy suggesting that there was a decline in smokers' perceptions of the harms of smoking in the latter part of our study period, we also examined the number of TVRs by campaign type for the final 2 years of the study period 9.

Finally, we calculated the percentage of TVRs across the study period that related to testimonial-style advertisements with negative health effects messages which, based on the existing evidence, seem likely to have been the most effective 1.

We did not feel it appropriate to conduct formal statistical analyses of the TVR data to assess if apparent trends over time were systematic or due in part to random variation. This was because we only wished to describe what the TVR data indicates, and also because we did not feel confident in attributing distributional properties to these data. Therefore, the various trends and differences that we suggest as systematic effects are based only on our observations of the data.

## Results

**Figure 1:**
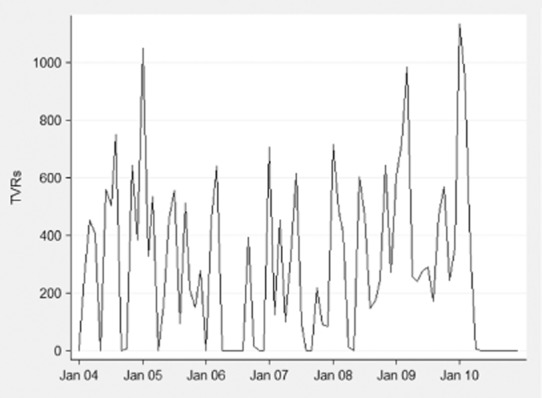
Tobacco control Television Ratings (TVRs) 2004–2010

A total of 24 507 tobacco control TVRs were broadcast during the study period. Figure 1 shows tobacco control TVRs during the study period. There was no discernible long-term trend in tobacco control TVRs. TVRs tended to peak in January and were highest in January 2005 and 2010.

**Figure 2:**
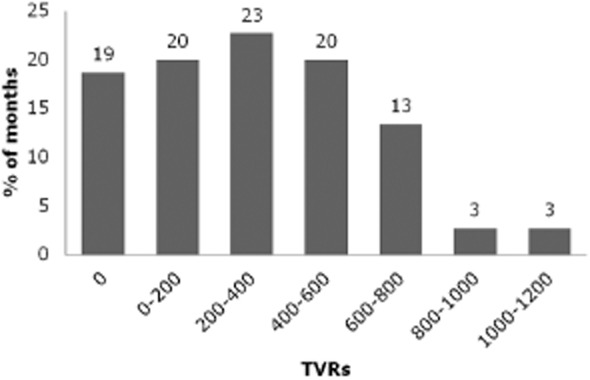
Intensity of campaigns, 2004–2010. Percentages add to 101 due to rounding

Between 2004 and April 2010 there were, on average, 3800 tobacco control TVRs per year, thus people were exposed to advertisements 38 times on average. Nineteen per cent of months had no tobacco control advertising, 43% of months had tobacco control campaigns but fewer than 400 TVRs, while only 39% of months had more than 400 TVRs, the level shown to be required to reduce smoking prevalence 2 (Fig. 2). A very small proportion of TVRs, 0.3%, could not be categorized because the films of these campaigns were not available.

During the study period the vast majority of TVRs (89%) were for adult cessation; in 2008–2010, this figure was 98% (Table 3). A very small proportion was for smoking in pregnancy (0.3%). Passive smoking campaigns made up 8% of all campaign exposure and were concentrated mainly in 2005 and 2007. Advertisements about smoke-free legislation made up 3% of TVRs during the study period; these advertisements were run exclusively in May and June 2007, before the legislation was implemented in July 2007. There were no TVRs for adolescent cessation, adult uptake or anti-industry advertisements during the study period.

**Table 3:** Key themes of campaigns, 2004–2010 [no. and % of Television Ratings (TVRs)]

		Total TVRs	Not categorized^a^ (%)	Key theme				
				Adult cessation (%)	Child uptake (%)	Pregnancy (%)	Passive smoking (%)	Smoke-free (%)
Full period (4 January–10 March)	TVRs	24 507	85 (0.3)	21 788 (88.9)	17 (0.1)	68 (0.3)	1862 (7.6)	687 (2.8)
8 April–10 March	TVRs	10 227	51 (0.5)	10 060 (98.4)	0 (0)	68 (0.7)	47 (0.5)	0 (0)

No TVRs for adolescent cessation, adult uptake, anti-industry during study period. ^a^Campaign creative not available.

As shown in Table 4, 46% of all advertising during the study period warned of the negative consequences of smoking. Forty-eight per cent contained information about how to quit using particular methods, primarily advertising local Stop Smoking Services; more than 60% of advertisements were of this type in 2008–2010. During this latter period only a quarter of advertisements warned of the negative consequences of smoking.

**Table 4:** Information content, emotional content and style of campaigns, 2004–2010 [number and % of television ratings (TVRs)]

		Total TVRs	Not categorized^a^ (%)	Informational content			Emotional content		Style				
				Negative consequences of smoking (%)	Positive consequence of quitting (%)	How to quit (%)	Evoke negative feelings about smoking (%)	Evoke positive feelings about quitting (%)	Acted scenes (%)	Testimonial^b^ (%)	Graphic aid (%)	Features music (%)	Features a child (%)
Full period (4 January–10 March)	TVRs	24 507	85 (0.3)	11 250 (45.9)	1295 (5.3)	11 849 (48.4)	12 256 (50.0)	10 914 (44.6)	17 744 (72.4)	4064 (16.6)	2696 (11)	12 741 (52.0)	7650 (31.2)
8 April–10 March	TVRs	10 227	51 (0.5)	2692 (26.3)	1295 (12.7)	6275 (61.4)	3853 (37.7)	6276 (61.4)	8439 (82.5)	611 (6.0)	1176 (11.5)	8227 (80.4)	4999 (48.9)

No TVRs featuring cartoons during study period. Multiple answers could be given for information content, emotional content and style of delivery. ^a^Campaign creative not available. ^b^All testimonial advertisements featured negative health effects messages. This category therefore reflects the advertisements expected to have been most effective during the study period.

Half of all TVRs during the study period evoked negative emotions, including all those which warned of the negative consequences of smoking (Table 4); 45% evoked positive emotions.

Seventy-two percent of advertisements during the study period were acted (Table 4). Testimonial-style advertisements, featuring real-life smokers and/or their friends or relatives, accounted for 17% of campaign exposure during the study period. A third of advertisements featured children, either as actors or giving testimonials. All the testimonial advertisements contained negative health effects messages and evoked negative emotions.

## Discussion

This study found that the majority of government-run tobacco control campaigns in England between 2004 and 2010 were for smoking cessation. Nearly half warned of the negative consequences of smoking. Forty-eight per cent contained information about how to obtain smoking cessation support; 61% contained this information in 2008–2010. Most advertisements featured acted scenes, and only a small proportion were testimonial advertisements. Between 2004 and 2010, in most months, there were fewer than four exposures to tobacco control advertisements per head.

To our knowledge, this is the first study to characterize tobacco control MMC in England. By applying a single framework to all advertisements, we have ensured consistency in our characterization. A potential weakness is that our coding was conducted by only two researchers, and may therefore be subject to bias. However, the majority of elements in our framework are objective and, furthermore, comparing our coding with that of a group of smokers suggests that their interpretations were highly comparable to ours. The focus group also suggested that there are no major themes or categories omitted from the framework. A further limitation is that we have looked only at advertisements funded by the Department of Health which were shown on TV, rather than on radio, in the press, etc. However, TV advertising accounted for approximately two-thirds of tobacco control advertising spend during the study period, and our results should therefore be representative of overall advertising exposure (Central Office of Information media plans, personal communication).

Our data did not cover all the tobacco control campaigns that were run in England during the study period. There were some local and regional-level campaigns which are likely to have been targeted more closely to local needs. In addition, there were several campaigns funded by the Department of Health and run by well-known charities (British Heart Foundation and Cancer Research UK): the Smoke Is Poison Campaign (Cancer Research UK), the Fatty Cigarette Campaign and the Under My Skin Campaign (both British Heart Foundation). These campaigns made up only a small proportion of campaign exposure during the study period, accounting for fewer than 1200 TVRs combined (British Heart Foundation and Cancer Research UK media plans, personal communication). These were, however, exclusively hard-hitting campaigns warning of the health risks of smoking and therefore may have had an impact on smoking behaviour during the study period.

Our findings suggest that although many elements of recent tobacco control campaigns are likely to have been effective, based on existing recommendations from the literature MMCs in England may not have been maximally effective.

Previous studies suggest that 400 TVRs per month are required to reduce smoking prevalence 1. Between 2004 and 2010, 43% of months did not reach this threshold, indicating that the intensity of campaigns has often been insufficient. In addition, prior to 2008, there were many months (19%) with no campaigns at all, demonstrating that campaigns were often not sustained, even though evidence suggests that their effects only last for 2–3 months 2,4,5.

Existing evidence suggests that negative health effects messages are effective; there is less evidence in support of how-to-quit messages 1. The extensive use of how-to-quit messages in campaigns in England could therefore be misguided. There is some evidence that highly emotional and/or testimonial-style negative health effects messages are the most effective at driving quitting behaviour 10,11. This implies that the high proportion of acted how-to-quit messages in recent years may have reduced the effectiveness of campaigns. All testimonial advertisements during the study period gave information on the negative consequences of smoking and evoked negative emotions; these may have been the most effective, yet accounted for only 17% of total TVR exposure. This could be due partly to the fact that emotionally engaging testimonial advertisements dealing with the negative consequences of smoking are difficult to produce. They require people who have a smoking-related condition (or their friends and relatives), who have a strong appeal to other smokers and who are willing to participate. These advertisements are generally unscripted and therefore time-consuming to record, and their production may be particularly difficult if the subject of the interview is unwell.

The types of campaigns identified in this study were in line with objectives highlighted in the White Paper ‘Choosing Health’, published in 2004, which proposed that mass media campaigns should contain information about the health risks of smoking and reasons not to smoke, as well as information about how to access support to quit smoking 12. Our characterization suggests a shift towards more how-to-quit messages later in the study period; this is likely to be a result of a marketing strategy developed in 2007–08. The objectives of this strategy were to trigger action (for example, by encouraging quitline calls), make quitting easier [by using National Health Service (NHS) support] and to reinforce motivation. Informing people of the health risks of smoking was not included as an objective 13. Policy makers have acknowledged that the shift towards a higher proportion of how-to-quit messages following the implementation of smoke-free legislation in England may have had a detrimental impact on motivation to quit. This has been highlighted in the recent tobacco control marketing strategy, which outlines future plans to run campaigns which simultaneously reinforce motivation to stop and direct smokers to effective cessation support or information 9.

This study has highlighted potential strengths and limitations of tobacco control MMCs in England. Based on international evidence on which MMCs are likely to be most effective, it suggests that public funds might not be being spent as effectively as possible. There is, however, no evidence of the differential effectiveness of campaigns specific to the United Kingdom and further research in this area is therefore required to maximize the impact of forthcoming campaigns in the United Kingdom. Furthermore, evidence on the relative effectiveness of different elements of successful campaigns, such as negative health effects messages and testimonial-style advertisements, is required. Finally, future studies should investigate the impact of campaign features which have not been studied elsewhere, such as the effect of second-hand smoke campaigns. The TVR data and the coding framework used in this study provide a starting-point for future studies. In ongoing research they are being used in conjunction with survey and routinely collected data containing information on smoking and quitting behaviour to investigate the impact of mass media campaigns during the study period.

### Declaration of interests

Tessa Langley and Michelle Sims are funded by the National Prevention Research Initiative www.mrc.ac.uk/npri (Grant number MR/J00023X/1). The Funding Partners relevant to this award are: Alzheimer's Research Trust; Alzheimer's Society; Biotechnology and Biological Sciences Research Council; British Heart Foundation; Cancer Research UK; Chief Scientist Office, Scottish Government Health Directorate; Department of Health; Diabetes UK; Economic and Social Research Council; Health and Social Care Research and Development Division of the Public Health Agency (HSC R&D Division); Medical Research Council; The Stroke Association; Wellcome Trust; and Welsh Assembly Government. Robert West has undertaken research and consultancy for companies that develop and manufacture smoking cessation medications. He has a share of a patent in a novel nicotine delivery device. His salary is funded by Cancer Research UK. None of the other authors declare any potential financial conflicts.
